# The impact of area level mental health interventions on outcomes for secondary school pupils: Evidence from the HeadStart programme in England

**DOI:** 10.1016/j.econedurev.2023.102425

**Published:** 2023-10

**Authors:** Sarah Cattan, Suzet Tanya Lereya, Yeosun Yoon, Ruth Gilbert, Jessica Deighton

**Affiliations:** a Institute for Fiscal Studies and IZA, United Kingdom; b University College London and Anna Freud, United Kingdom; c University College London, United Kingdom

**Keywords:** Exclusion, Absenteeism, Mental health, Intervention, Adolescents, Synthetic control methods

## Abstract

In light of the dramatic rise in mental health disorders amongst adolescents seen in the past decade across the world, there is an urgent need for robust evidence on what works to combat this trend. This paper provides the first robust evaluation of the impacts on school outcomes of 6-year funding programme (*HeadStart*) for area-level mental health interventions for adolescents. Exploiting educational administrative data on ten cohorts of state-educated secondary school students, we use the synthetic control method to construct counterfactual outcomes for areas that received the funding. We show that the funding did not affect students’ absenteeism or academic attainment, but it prevented around 800 students (c. 10% of students typically excluded yearly) from being excluded in its first year. The transient nature of this effect suggests that sustained funding for intervention may be a necessary but not sufficient condition to maintain programme effectiveness over time.

## 1 Introduction

Mental illness has been ranked first in terms of global burden of disease, accounting for just under a third of the years lived with disability (Vigo, Thornicroft & Atun, 2016). Adolescence is a critical period for young people's health and wellbeing (Dahl, Allen, Wilbrecht & Suleiman, 2018; Patton et al., 2016), as half of mental ill health starts by age 15 and three quarters by age 24 (Kessler et al., 2004). Mental health disorders experienced in adolescence have a wide-range of impacts and implications both within adolescence and adulthood, including educational outcomes. Prospective longitudinal studies have reported associations between emotional and behavioural problems and subsequent academic achievement (Deighton et al., 2018; Masten et al., 2005; Moilanen, Shaw & Maxwell, 2010) as well as higher rates of subsequent absenteeism (Kearney, 2008) and exclusions from school (Lereya & Deighton, 2019; Paget et al., 2018; Parker, 2014; Whear et al., 2013).

The past few decades have seen a significant rise in mental health disorders amongst adolescents. In the UK, the setting of this study, one in eight children and young people report problems that meet standardised definitions of mental disorder, with this proportion rising to one in six during the COVID-19 pandemic (NHS Digital, 2020). Recent escalations in mental health problems in children and young people have raised the government's interest in providing programmes that embed mental health prevention and early intervention support in schools and communities. Current examples, in England, include the 2017 Green Paper: *Transforming children and young people's mental health provision*, which channels significant resource into building capacity for training mental health leads in schools and the introduction of mental health support teams for schools that are overseen by specialist mental health staff. Such approaches require significant national and local investment and are often rolled out in a way that varies across local areas and different contexts, making summative evaluation challenging. If we are to discern whether such approaches have the desired impact, it is important that we find ways to explore their potential benefits whilst navigating their complex and variable delivery.

There are both theoretical reasons and empirical evidence to believe such approaches can be effective. On the one hand, area-level approaches are supported by ecological models of child development (Bronfenbrenner, 1992) and resilience (Masten & Cicchetti, 2016), which highlight that mental health and wellbeing are affected by multiple levels of the systems surrounding the child, including families, schools and neighbourhood. On the other hand, recent empirical evidence suggests that focusing significant resources on providing prevention and early intervention within school settings may be an effective way to support children and young people's wellbeing (Clarke et al., 2021; Lereya, Ullman, Testoni, Wolpert & Deighton, 2019; Pilling et al., 2020), and that such universal and targeted support is more effective when it is embedded in whole school and whole systems approaches (Melendez-Torres, Allen, Viner & Bonell, 2022). Furthermore, where these interventions reach beyond the school to incorporate community elements, they achieve greater impact, particularly for social and emotional outcomes (Goldberg et al., 2019).

Despite such evidence of promise, there is still limited robust causal evidence about the effectiveness and cost-effectiveness of these types of approaches on the outcomes of young people (Public Health England, 2015). Evaluating this type of intervention can be challenging for a number of reasons. First, it can be challenging to find a suitable comparator group for intervention areas. So far, funders have had little appetite for random allocation of these area level interventions because of the costs, forward planning, coordination, processes for consent and time required for RCTs. The areas that do receive funding often have particularly poor outcomes at baseline or a local system that is particularly interested in mental health prevention, making them systematically different from other areas in aspects that are not easy to measure and control for in an evaluation. Second, it can be difficult to find suitable data to conduct such evaluation. Indeed, even if a suitable comparison group could be found, the data requirements for this type of evaluation can be high. Large sample sizes are needed to detect area-level Intention-To-Treat effects expected to be relatively small. Data on mental health outcomes are usually not routinely collected in schools and/or communities, and large-scale primary data collection can be difficult to implement, especially in comparator areas who see less incentive to engage in burdensome data collection.

The contribution of this paper is to provide evidence of the causal impact of funding an area-level programme called HeadStart on school absence, exclusion and attainment of secondary school pupils in England, three outcomes the programme aimed to affect through mental health improvement. This model epitomises some of the challenges often faced in the evaluation of area level interventions in that it was implemented across a range of different areas with different challenges and resources; the intervention includes a range of contexts and multiple interventions nested in a wider ecological framework; it also varies significantly from area to area in content and implementation, although the aims and key principles are consistent across areas. We overcome the evaluation challenges described above by implementing the synthetic control method in nationally collected, longitudinal administrative data on all secondary school pupils educated in mainstream state schools.

In 2016, the National Lottery Community Fund provided six local authorities (LAs) with c. £10 m each to design and implement new interventions aiming to promote young people's mental health, wellbeing, and resilience over a period of 6 years. Each LA used the funding to offer a different bundle of interventions primarily intended to increase wellbeing and reduce onset of mental health problems which also aimed to benefit academic outcomes in the long-term. In this context, we propose to identify the Intention-To-Treat effect of providing the funding to the six HeadStart LAs on the outcomes of students attending schools in these LAs. This parameter is of high policy relevance because it is crucial to compute the economic returns from this investment.

The 6 LAs that received HeadStart funding were selected non-randomly through a relatively opaque process, which does not provide a natural control group. To circumvent this challenge, we use the synthetic control method developed by Abadie and Gardeazabal (2003) and extended by Kreif et al. (2016) to the case of multiple treated units. For each outcome, we create a synthetic control group for the HeadStart LAs by estimating weights for each of the 143 Local Authorities that did not receive funding for the programme (the donor pool) such that the weighted average of the outcome in these areas (the synthetic control group) mimics the average outcomes of pupils in the treated areas as closely as possible in the pre-programme period. For all outcomes of interest, the synthetic control group approximates the outcome pre-trends very well, which gives confidence that the outcomes of the synthetic control group in the programme period provides a good counterfactual for the outcomes of the treated areas and that the programme impact can be measured as the difference in outcomes between the HeadStart areas and the outcomes of the synthetic control group.

We focus on three types of outcomes: school absence and school exclusions throughout secondary school, and attainment in Year 11 (age 16). Using data on up to 11 academic years, we measure these outcomes using individual-level data on all students attending mainstream schools in the 6 HeadStart LAs (*n* = 1729,646) and in the other 143 LAs in the donor pool (*n* = 28,501,357). To perform the synthetic control method, we aggregate them up to the LA and academic year level. For absence and exclusion, we use data between 2008/09 and 2018/19, and for attainment, we use data between 2014/15 and 2018/19. HeadStart started in academic year 2015/16, so when we look at absence and exclusion as outcomes, the synthetic control group was created based on seven years of data (from the pre-programme period). When we look at attainment as outcomes, the synthetic control group was created based on three years of data (from the pre-programme period). Because the interventions were developed over time and impacts could take time to materialize, we estimate dynamic impacts of the funding on outcomes measure in each of the three years following the start of the programme.^1^ amongst the outcomes considered, the only effect we are able to detect is a transient reduction of the incidence of exclusion (or suspension) amongst secondary school students (aged 10–16y) of marginal statistical significance. By 2 years after the start of the HeadStart intervention, there was no measurable difference in exclusions between HS- and Non-HS LAs. This reduction seemed to be driven by both a reduction in the incidence of fixed term and permanent exclusions. In contrast, we did not find a significant effect on the proportion of sessions missed due to a (fixed term) exclusion, which may indicate that HeadStart prevented students on the margin of exclusion from being excluded rather than actually reduced the length of exclusion for those students with more severe reasons to be excluded. Moreover, the fact we found no comparable effects on absences and age 16 attainment might be driven by a change in school's approach to disciplinary practices rather than a change in young people's behaviour. Furthermore, HeadStart programme might have improved behaviour difficulties more than other mental health difficulties which might have reduced the rates of behavioural outbursts which might have led to exclusions

The rest of the paper is organised as follows. We provide some background on HeadStart in Section 2. The empirical strategy and data are described in Sections 3 and 4, while results are discussed in Section 5. Section 6 concludes with a discussion.

## 2 Background on the headstart programme

Started in 2016, HeadStart was a six-year, £67.4 million National Lottery funded programme set up by The National Lottery Community Fund. The programme aimed to explore and test new ways to improve the mental health and wellbeing of young people aged 10 to 16 and prevent serious mental health issues from developing. Considering the impact of mental health difficulties on absenteeism (Panayiotou, Finning, Hennessey, Ford & Humphrey, 2021) and academic attainment (Deighton et al., 2018; Panayiotou & Humphrey, 2018), by improving mental health and wellbeing of young people, the programme also aimed to increase academic outcomes over the long-term.

To do so, HeadStart formed six partnerships in six different local authorities (LAs) in England: Blackpool, Cornwall, Hull, Kent, Newham, and Wolverhampton. Each partnership received approximately £10 m over 6 years from Sept to August 2015/16 to 2021/22 to deliver a range of mental health interventions for 11–16-year-olds attending schools in the LA.^2^ Wolverhampton started delivering interventions one year after the others, from academic year 2017/18, in order to spend more time on development before delivery. We take this into account in our estimation of treatment effects by not including Wolverhampton in the treated unit in 2016/17.

Between 2016 and 2022, each partnership worked with young people in their local area broadly in the target age range (10–16 years), schools, families, charities, community and public services to form a variety of interventions that best fit the needs of their local communities. Some of these interventions were child focused (e.g., peer mentoring, one-to-one counselling), some were directed at school staff or other professional groups, and some were directed at parents and carers. Accordingly, they were delivered in a range of settings including schools, communities, families, and the digital world, though the majority took place in schools.

Interventions were reported to have one of the following four aims: increasing mental wellbeing, improving school engagement, reducing mental health difficulties, and reducing risky behaviour. Interventions delivered can be clustered into five broad types. Table 1 describes the five main types of interventions delivered within the 6 HeadStart local areas and provides examples of specific interventions within each type. More information about the interventions can be found on HeadStart briefings (HeadStart Learning Team, 2019; Lereya, Edridge, Nicoll & Deighton, 2022).

**Table 1:** HeadStart interventions description.

Intervention type	Description	Examples
Whole school wellbeing promotion interventions	Preventative interventions to build resilience and prevent mental health difficulties	Whole school training School-based resilience approaches
Child focused targeted interventions to improve mental health difficulties and wellbeing (Individual or group sessions)	Interventions accessed by young people who are experiencing mental health difficulties or who are at greater risk of developing difficulties in the future	Building skills based on cognitive behavioural techniques with small groups Community based targeted group work
School staff training or supervision	Staff training to spot signs of mental health issue and prevent mental health issues from getting worse	Youth Mental Health First Aid Training Diploma in Trauma and Mental Health Informed Schools
Parent/carer interventions	Interventions to help parents provide emotional warmth, stability and consistency	Parent peer mentor project Empowering Parents, Empowering Communities: Being a parent course
Online support	Interventions that were delivered online to improve mental health and wellbeing	Online counselling

Each HeadStart partnership was free to choose interventions they saw as best fitting the needs of their setting and their young people. Moreover, they were free to choose whether to intervene in all or a subset of secondary schools. Accordingly, in this evaluation, we focus on identifying and estimating the effect of providing the funding to deliver a locally selected bundle of interventions. We formally define our treatment in Section 3 below.

## 3 Empirical strategy

Our main analysis focuses on estimating the causal effect of offering HeadStart funding to Local Authorities on student school outcomes. The main analysis is therefore at the Local Authority level and focused on recovering an Intention-To-Treat (ITT) parameter. This parameter is highly policy relevant as it focuses on evaluating the benefits from the funding received, taking into consideration the fact that LAs were relatively free to use this funding in ways that best fitted the needs of their population. It is also an important parameter to estimate to compute the economic return to the funding.

### 3.1 Overview of the synthetic control method

Given our focus on the LA-level ITT and the fact that six LAs received HeadStart funding, we follow Kreif et al. (2016) to implement the synthetic control method in the context of multiple treated units. Denote *A* the total number of units (Local Authorities) in our sample, with $A_{0}$ treated units and $A_{1}$ non-treated units. We refer to these non-treated units as the donor pool. For each Local Authority *j*, we observe the outcome vector $Y_{j}=\left(Y_{j1}\ldots Y_{jT_{0}}\ldots Y_{JT}\right)$ for T academic years, where $Y_{\text{jt}}$ is the outcome in Local Authority *j* and time period *t*, and denote the year in each Headstart funding started $T_{0}$+1 (because we have three treatment years, $T_{0}$+1 *=* *T – 3*).

The data generating process for each outcome for each local authority *j* and academic year *t* can be written as the sum of a treatment-free potential outcome, $Y_{jt}^{N}$, and the effect of the average treatment effect (ATE) $\alpha_{jt}$, such that:

$$Y_{jt}=Y_{jt}^{N}+\;\alpha_{jt}D_{jt}$$

$$Y_{jt}=\delta_{t}+\lambda_{t}\mu_{j}+\theta_{t}Z_{j}+\epsilon_{jt}+\alpha_{jt}D_{jt}$$

where $\delta_{t}$ is an academic year fixed effect, $Z_{j}$ is a vector of time-invariant predictors with time-varying coefficient vector $\theta_{t}$, and $D_{jt}$ an indicator for treatment that takes the value 1 if the Local Authority received the HeadStart funding and 0 otherwise.

For each year of the pre-intervention period, the treatment-free potential outcome $Y_{jt}^{N}$ corresponds to the observed outcome, for both the treated and the control regions. For periods during which the programme is implemented, the treatment-free counterfactual outcomes, $Y_{1t}^{N}$, were only observed in the control regions. The goal of the synthetic control approach is to estimate the unobserved counterfactual $Y_{1t}^{N}$ for the treated region by creating a `synthetic control unit’ that best approximates the relevant pre-intervention characteristics of the treated region. Formally, this synthetic control unit is obtained by estimating the vector of weights $W=(w_{2},\ldots ,\;w_{J+1}),$associated with each control region *j* that minimize the discrepancy in the observed and unobserved confounders measured before the intervention, between the treated and the synthetic control region and under the constraints that weights are all non-negative and sum to 1.

Following Abadie, Diamond and Hainmueller (2010), we minimize the following distance metric:

$$d\;=\;\sqrt{{\left(X_{1}-X_{0}W\right)}^{\prime }V\left(X_{1}-X_{0}W\right)}$$

where $X_{0}$ is k x 1 metric of covariates, including pre-treatment outcomes and predictor variables for the treated area and $X_{1}$ an equivalent *k x A* matrix for control areas. *V* is a *k* × k positive definite and diagonal matrix, which assigns weights according to the relative importance of the covariates and the pre-intervention outcomes. What variables go into X and the weight attached to them in the matrix V can be a subjective decision, justified by knowledge of the process driving outcomes. Abadie et al. (2010) propose choosing V and W in order to minimize the mean squared prediction error. This approach has largely been followed in the literature and is the one we follow here as well.

Once these weights are estimated, the treatment effect for the treated unit for each time period after $T_{0}$ can be obtained as $\hat{\alpha_{1t}}$= $\;Y_{1t}-\hat{Y_{1t}^{N}}\;$, which is the difference between observed outcomes in the treated regions and their counterfactual outcomes constructed as the linear combination of the observed outcomes of the potential control regions: $\hat{Y_{1t}^{N}}=\sum_{j=2}^{J+1}w_{J}Y_{jt}$.

### 3.2 Inference

Whilst there is no uncertainty in the value of the aggregate units, there is uncertainty in the extent to which they can capture the potential outcomes in the absence of treatment. Abadie et al. (2010) propose to capture this uncertainty through placebo tests, and we follow the modification of this method proposed by Kreif et al. (2016) to accommodate for the fact that we have 6 treatment units and that it is easier to construct synthetic controls for large regions than it is for small individual ones.

The procedure aims to construct a test for the null hypothesis that the treatment effect is 0. To obtain the distribution of treatment effects under the null, we build placebo-treated regions by re-sampling a set of LAs from the pool of LAs (including both treated and non-treated LAs), without replacement, keeping the remaining areas in the placebo donor pool. For each placebo-treated region *p*, we estimate a placebo-treatment effect, $\hat{\alpha_{jt}^{p}}$, using the synthetic control procedure outlined above. In this paper, we repeated this procedure 500 times to construct a distribution of treatment estimators for the treatment period under the null hypothesis of zero treatment effect. For each parameter of interest, we report the *p*-value of the two-sided test as the proportion of placebo average treatment effect $\hat{\alpha_{jt}^{p}}\;$that were at least as extreme in absolute value as the estimated $\hat{\alpha_{jt}}$.

## 4 Data

### 4.1 Data source and sample

The data used for analysis come from the National Pupil Database (NPD), which is an administrative dataset collected by the Department for Education (DfE) on all state-educated pupils in England born since 1986.^3^ This study uses data on pupil-level absenteeism records and exclusion records and pupil-level key stage 4 attainment scores (GCSEs), linked to the annual pupil census data in the NPD and census of children in social care (Children In Need and Children Looked After). We focus our analysis on students attending mainstream secondary schools.

Our analysis considers several outcomes measuring school absence, school exclusion and school attainment. For absence and exclusion outcomes, we use data for academic years 2008/09 to 2018/19. Given that HeadStart started in academic year 2016/17, this means that we have 8 years of pre-intervention data (2008/09 to 2015/16) and 3 years of intervention data (2016/17 to 2018/19). For attainment, we use GCSEs, which is a standardised measure of attainment in Year 11 (when students are 16/17 years old). We use three years of pre-intervention data (2013/14 to 2015/16) and 3 years of intervention data (2016/17 to 2018/19).^4^ We do not consider treatment effects beyond 2018/19 despite the fact that the funding continued because the COVID-19 pandemic from March 2020 affected the outcomes and data collection in England.

The donor pool includes 143 LAs that never received the HeadStart funding.^5^ As mentioned earlier, HeadStart funding was provided in 5 LAs from 2016/17 and one additional LA (Wolverhampton) received it from 2017/18. Therefore, to estimate the impact of HeadStart on outcomes measured in 2016/17, we average the outcomes of pupils attending schools in the 5 LAs who had received HeadStart funding by then and compared them to the outcomes of pupils in the synthetic control group. To estimate the impact of HeadStart outcomes measured in 2017/18 and 2018/19, we average the outcomes of pupils attending school in the 6 LAs who had received HeadStart funding by then and compared them to the outcomes of pupils in the synthetic control group.^6^

Our analysis is conducted at the LA and academic year level. To estimate the weights attributed to each LA in the control pool, we included in the vector $X_{0}\;$and $X_{1}$ the outcome of interest in each of the pre-treatment years, as well as the demographic characteristics averaged across all pre-treatment years. We provide further details on our outcomes and covariates below.

### 4.2 Outcomes

We evaluate the impact of HeadStart funding on five primary outcomes defined at the LA and academic year level, measuring absence, exclusion and attainment in secondary school. We construct these outcomes at the individual level and then aggregate them by taking their means at the LA and academic year level in our analytical sample. Panel A of Table 2 reports the mean of these five outcomes in the underlying, individual-level data across the pre-intervention period used in the analysis (2008/09 to 2015/16 for absence and exclusion; 2013/14 to 2015/16 for GCSEs). The first column reports these means across the full sample of mainstream schools in England, while the second and third columns show them for the Local Authorities that received and did not receive HeadStart funding respectively.

**Table 2:** Means of outcome and control variables in the pre-intervention period.

	Full sample	Non-Headstart LAs	HeadStart LAs
**Panel A - Pupil outcomes**			
Fraction all sessions missed for authorised reasons	0.047	0.047	0.048
Fraction all sessions missed for unauthorised reasons	0.013	0.013	0.015
Fraction all sessions missed for fixed term exclusion	0.001	0.001	0.002
Fraction pupils ever excluded	0.048	0.048	0.052
Fraction pupils with fixed term exclusion	0.001	0.001	0.001
Fraction pupils with permanent exclusion	0.048	0.048	0.052
Fraction pupils with at least 5 GCSEs A*-C	0.626	0.628	0.588
Fraction pupils with at least 5 GCSEs A*-C incl. Eng and Math	0.560	0.562	0.533
**Panel B - Pupil characteristics**			
Female	0.493	0.492	0.495
FSM	0.153	0.152	0.170
White	0.796	0.796	0.808
Black	0.048	0.048	0.046
Asian	0.073	0.073	0.072
Chinese	0.004	0.004	0.003
Other ethnic group	0.079	0.079	0.071
SEN	0.202	0.201	0.217
IDACI rank	47.484	47.826	41.881
Ever CIN	0.032	0.032	0.042
Ever CLA	0.003	0.003	0.003
*Number of individual level observations*	*21,943,361*	*20,682,388*	*1260,973*
*Number of LAs*	*149*	*143*	*6*

Note: Table reports the means of outcome and control variables used in the analysis for the years preceding the implementation of HeadStart. For all variables but the two GCSE outcome variables, this corresponds to academic years 2008/09 to 2015/16. For the two GCSE outcome variables, this corresponds to 2013/14 to 2015/16.

For absence, we create two outcomes at the individual level: the proportion of all sessions in the school year the student missed because of authorised absence(s) and the proportion of all sessions in the school year the student missed because of unauthorised absence(s). Authorised absences are primarily due to sickness and a small proportion of authorised absences is due to authorised holidays during term time. Unauthorised absences are due to unauthorised holiday during term time and other reasons (e.g. truancy). As shown in Table 2, secondary school students miss almost 4.7% of all sessions due to authorised absences and 1.3% of all session due to unauthorised absences. Absence rates are slightly higher amongst students who go to school in the LAs that received HeadStart funding on average across the 8 years prior to the intervention.

Student exclusion from school in England can be either for a fixed period or permanently, and it is not uncommon that students have one or several fixed period exclusions before being permanently excluded from school. We create two outcomes at the individual level: the proportion of all sessions in the school year the student missed because of fixed period exclusion, and a binary indicator that takes the value 1 if the student was excluded from the school (either for a fixed period or permanently). Permanent exclusions represent a very small proportion of all exclusions. In the pre-intervention period that we consider, 0.1% of secondary students are permanently excluded while 4.8% of them experienced at least one fixed period exclusion. amongst them, on average, secondary students miss 0.1% of all sessions because of a fixed term exclusion. As with absence, exclusion rates are higher in the HeadStart funded LAs than in the other LAs. Our main results focus on these two outcomes, but in the Appendix, we also report impacts on the proportion of students excluded for fixed periods and on the proportion of students excluded permanently separately.

Finally, we consider attainment at the end of Year 11, measured by GCSEs. Specifically, we define our outcome as a binary indicator that takes the value 1 if the student obtained 5 GCSEs A*-C (In the Appendix, we also report impacts on a binary indicator that takes the value 1 if the student obtained 5 GCSEs A*-C, including English and Math, which is another often used measure of attainment). GCSEs are standardised achievement tests taken by all students at the end of Year 11, and obtaining an A* to C grade on at least 5 GCSE subjects is a widely used threshold for good achievement in the English education, which has been shown to significantly affect later outcomes including university degree completion (Machin, McNally & Ruiz-Valenzuela, 2020).^7^

We run the synthetic control method on a dataset at the Local Authority and academic year level. To construct this dataset, for absence and exclusion, we average the outcomes in the individual level data across all year 7 to 11 students attending school in each LA and each academic year. For attainment, we average the outcomes in the individual level data across all year 11 students attending school in each LA and each academic year.

### 4.3 Covariates

We also include in the vector X a set of pupil characteristics. At the individual level, these covariates are defined as follows: an indicator for pupil gender, indicators for pupil ethnicity (Asian, Black, Chinese, Mixed, White or any other ethnic group), an indicator for whether the pupil is eligible for Free School Meal (FSM), an indicator for whether the pupil has special educational need (SEN) in the academic year, an indicator for whether the pupil was ever observed in the Child In Need (CIN) census and an indicator for whether the pupil was ever observed in the Child Looked After (CLA) census.^8^ Finally, we also use pupil's rank in the Income Deprivation Affecting Children Index (IDACI) based on the pupil's Lower Super Output Area of residence.^9^

Panel B reports the averages of these variables across all secondary students in all/HeadStart and non-HeadStart LAs in the pre-intervention period (2009–2016). During this period, the Local Authorities where HeadStart funding was provided were more deprived, with 17% of all students being eligible for FSM in HeadStart funded LAs vs. 15.20% in non-HeadStart funded LAs. The HeadStart funded LAs were also more ethnically diverse than the non-HeadStart funded LAs and had higher rates of pupils with Special Education Needs and with experience of children's social care system.

## 5 Results

**Fig. 1:**
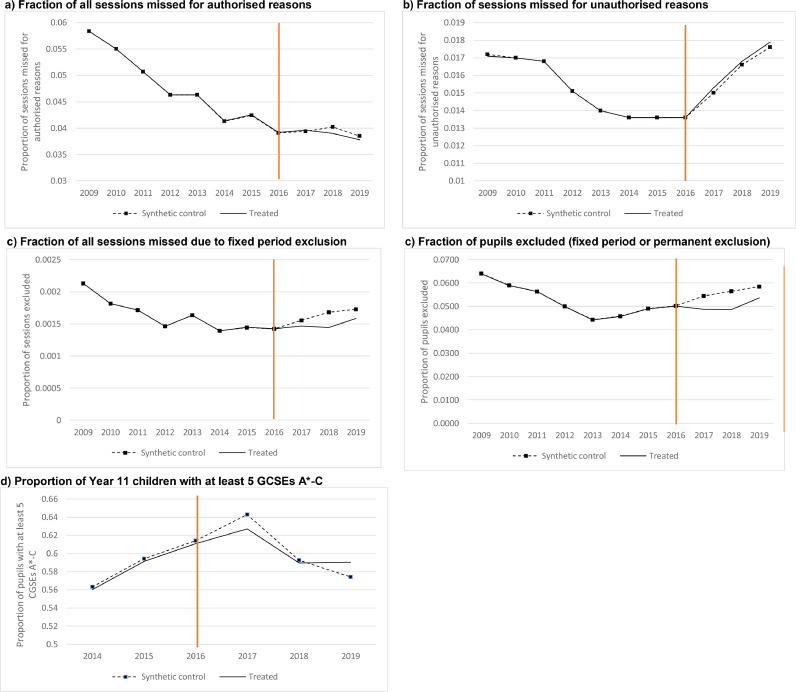
– Trends in outcomes of interest in the treated and synthetic control groups in the pre-intervention and intervention periods.

Note: In each figure, the black line plots the average outcome in the treated (HeadStart) LAs and the dashed line plots the average outcome in the synthetic control group. The red vertical line reports the last pre-intervention year. Each figure corresponds to a different outcome named at the top of the graph.

Fig. 1 reports the trends in our five main outcomes in the treated group (i.e. HeadStart funded Local Authorities) (solid line) and in the synthetic control group (dashed line) for all years between 2009 (2014 for age 16 attainment) and 2019. The synthetic control group outcome is computed as the weighted average of outcomes from all 144 potential local authorities in England, where the weights are computed to minimise the root mean squared prediction error (RMSPE) between the treated unit and the synthetic controls in the lagged outcome over the pre-intervention period.

In the pre-intervention period, the solid and dashed lines are very close to each other, which reflects the fact that for all outcomes, the estimated synthetic control unit is an excellent comparator group to the treated unit. This is confirmed by the fact that for all outcomes, the RMPSE, which is effectively a measure of how close our predictions come to capturing the true changes in the outcomes before HeadStart was rolled out, is extremely small (see 4th column of Table 3). While the RMSPE is our main measure of how well the synthetic control matches the treated unit pre-intervention, it is also useful to look at how balanced a wider set of characteristics, which predict levels and trends in outcomes, are between the synthetic control and the treated unit. Appendix Table A1 reports the means of these characteristics and confirm the fact that there is very good balance for all outcomes.

**Table 3:** HeadStart impact on main outcomes.

	Impact of Headstart			RMSPE
	2016/17	2017/18	2018/19	
Fraction of sessions missed for authorised reasons	0.0002714	−0.0012189	−0.0007557	2.56E-14
	*(0.44)*	*(0.13)*	*(0.26)*	
Fraction of sessions missed for unauthorised reasons	0.0002474	0.0002022	0.0002964	1.40E-14
	*(0.35)*	*(0.45)*	*(0.41)*	
Fraction of sessions missed b/c fixed term exclusion	−0.0000891	−0.0002391	−0.0001438	3.09E-14
	*(0.38)*	*(0.24)*	*(0.37)*	
Fraction of pupils excluded	−0.0056738	−0.0077867	−0.0047789	7.75E-14
	*(0.08)*	*(0.11)*	*(0.24)*	
Fraction of pupils with at least 5 GCSEs A*-C	−0.0159674	−0.0029881	0.0162614	4.98E-14
	*(0.07)*	*(0.48)*	*(0.11)*	

Note: This first three columns of the table report the estimate of the impact of HeadStart funding and their pvalue (in parenthesis) in each of the first three years of implementation. The fourth column reports root mean squared prediction error (RMSPE).

For each outcome, the difference between the solid and dashed lines in years 2017, 2018 and 2019 measure the causal effect of the HeadStart intervention on the particular outcome. Table 3 reports the estimates of such effects of each outcome and each intervention year. To get a sense of how large these effects are, we can compare these effect sizes to the pre-intervention period averages reported in Table 2.

As can be seen in both Fig. 1 and in Table 3, there is almost no difference in the proportion of sessions missed due to either authorised or unauthorised absences between the intervention and control groups in the years 2017 onwards. However, there is a marked reduction in the exclusion rates of secondary school pupils in these years in the HeadStart LAs relative to the synthetic control made of non-HeadStart LAs. This reduction is larger in 2016/17 (0.6 percentages points, ppt) and 2017/18 (0.8 ppts) than it is in 2018/19 (0.5 ppts) and represents between a 10 and 15% relative reduction in the exclusion rate in the local authorities that received Headstart funding on average. Turning to Year 11 attainment (GCSEs), the estimates point to a reduction in the proportion of students achieving 5 GCSEs A*C in 2016/17 of 0.016 percentage points, which represents a 2.5% relative reduction in the pre-intervention period rate of students achieving such outcome. In 2017/18, the effect is very small but in 2018/19 the estimates point to an improvement in GCSE outcomes of approximately the same size as the worsening of outcomes in 2017/18.^10^

**Fig. 2:**
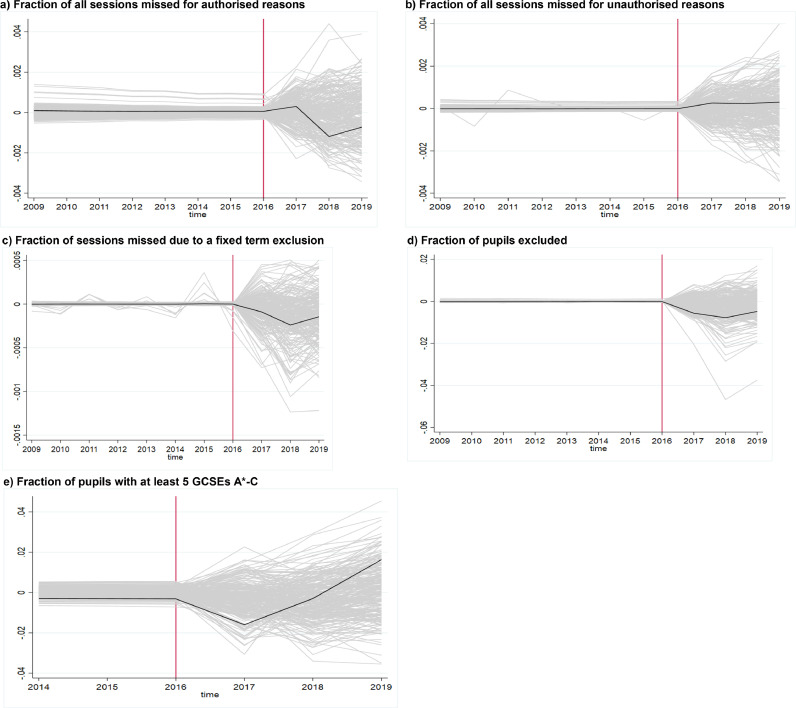
Gap in outcomes between HeadStart group and synthetic control (black) compared with the distribution of 250 placebo gaps (gray)

Note: In each figure, the black line plots the gap in one outcome between the HeadStart treated group and synthetic control group, and each grey line reports the gap in outcome between the placebo treated group and the relevant synthetic control group. We plot 250 placebo gaps (this is the maximum that Stata allows to plot). The red vertical line reports the last pre-intervention year. Each figure corresponds to a different outcome named at the top of the graph.

P-values obtained using Kreif et al. (2016) are reported below the point estimates in Table 3, and in Figure 2 we show the graphs showing 250 of the placebo tests underlying these p-values.^11^ The dark line shows the estimated difference between the true treated unit (Headstart unit) and its synthetic control, while each of the light grey lines shows the estimated treatment effect for each of the placebo-treated region. As can be seen from Fig. 2, for all outcomes but exclusion (Fig. 2c), the estimated differences in impact between the HeadStart treated unit and its synthetic control were well within the normal range of differences obtained by chance (under the null of no impact). For exclusion however, we see that the estimated differences in outcome between the HeadStart treated unit and its synthetic control was unusually small relative to the other placebo differences in the first two years of treatment, which gives greater confidence in the fact that HeadStart did play a causal role in reducing the incidence of exclusions in the Local Authorities where the programme was implemented. Based on these placebo tests, we compute p-values following Kreif et al. (2016) for each of the treatment effects reported in Table 2. Using this method, we find that the impact on exclusions in 2016/17 has a p-value of 0.08 and the impact on exclusions in 2017/18 has p-value 0.11.

### 5.1 Subgroup analysis

Impacts of the programme could vary across pupils with different characteristics. In order to explore this further, we re-estimate the model this time on subgroups defined by the following characteristics: male vs female, pupils with vs without a SEN statement, pupils who are vs who are not eligible for Free School Meals, and pupils who have vs have never been on a children's social care plan (Child Protection Plan or Child Looked After). The results of these analyses are reported in Appendix Table A3, Appendix Table A4, Appendix Table A5, Appendix Table A6. They suggest that reductions in exclusions may have been stronger for males and children who have been in care – potentially highlighting the fact that the policy was more effective amongst children who were more at risk of such behaviour to begin with. That being said, we would refrain from leaning too strongly on these conclusions as few impacts on subgroups are statistically significant and few differences in impacts between the two are also likely to be.

## 6 Discussion

Recent years have shown an increase of mental health problems amongst children and young people. This increase coincides with an increase of policy and practice focus in the UK in terms of implementing mental health support for both prevention and early intervention across schools and communities. Many complex area level interventions that aim to decrease mental health difficulties and increase wellbeing include a range of non-clinical and clinical programmes. When it comes to investigating the effectiveness of these complex interventions, researchers usually struggle to find comparable control groups. Hence, it is important to find methodologies that will enable better evaluation of complex area level interventions.

The primary contribution of this study is to estimate the impact that an area-level intervention aimed to decrease mental health difficulties and increase the wellbeing of adolescents in England (HeadStart) had on school outcomes such as attendance, exclusion and attainment.

Using nationally representative administrative education data, we show that the area level mental health intervention in England called HeadStart did not have a significant impact on absenteeism (authorised or unauthorised) or attainment. However, the intervention led to a transient decrease in exclusion rates across HeadStart funded Local Authorities in 2016/17 at the boundary of statistical significance (and even more so in 2017/18). The magnitude of these effects are non-negligible however: only in 2017/18, we estimate that HeadStart would have prevented around 800 pupils from being excluded (compared to a yearly average of around 7500 in the pre-intervention period).

The fact that the intervention decreased exclusion on the extensive rather than on the intensive margin, combined with the absence of effects of absence and attainment, suggests that these transient impacts on exclusions may have been driven by a culture change in schools’ approach to disciplinary measures as opposed an underlying change in pupil's behaviour. The interventions across HeadStart aimed to decrease mental health difficulties of children and young people *as well as* change the school environment to a more inclusive space with more identification and help to those in need. Previous research suggests that exclusion depends to an extent on the culture of the school, the resources, the needs of the staff, the wider community, social circumstances, the discipline policies of individual schools, the degree of tolerance maintained by different head teachers as well as the child's behaviour (Wright, Weekes & McGlaughlin, 2000).

Of course, it is not possible to completely rule out that the study itself, with increased monitoring and awareness of exclusion rates as a key metric, may have contributed to reduce exclusions – termed the Hawthorne effect – the effect of being studied, regardless of the intervention, though it would be harder to explain why impacts faded in the third year of implementation under this hypothesis. Instead, it is more likely that HeadStart struggled with sustainability due to diminishing enthusiasm towards the programme and staff turnover, a pattern that has been found to be true in many other mental health interventions (Moore, Stapley, Hayes, Town & Deighton, 2022). Going forward, this which would suggest that more is required from leaders and policymakers for mental health and wellbeing interventions to be successfully sustained.

Nevertheless, given the very high social cost associated with exclusions, even interventions that have a small impact on rates of exclusion can have large social benefits. Indeed, exclusions from school has been associated with poor academic progress in the short run but also poor prospects in terms of future academic attainment, employment and training.^12^ Exclusion from school have also been argued to cause long-term psychological illness as well as worsening of existing mental health illness (Gill, Quilter-Pinner & Swift, 2017; Parker et al., 2016; Whear et al., 2013) and increase the risk of self-harm (Parker et al., 2016). Lastly, there is also an established link between school exclusions and criminal activity (Williams, Papadopoulou & Booth, 2012) and recidivism (Williams et al., 2012). While most of the evidence suggesting these pathways between exclusions and these later outcomes are based on longitudinal studies rather than experimental or quasi-experimental designs, recent evidence by Bacher-Hicks, Billings and Deming (2019) do suggest there is likely to be a causal link between school exclusions and future criminal activity.

Current estimates of the individual and social cost of exclusions are high as a result. For the UK, the cost of a permanent exclusion is estimated to be close to £385,000 (HM Treasury, 2020), while we estimate that the cost of missing one session due to fixed term exclusion is estimate at close to £300 (all in 2019/20 prices).^13^ Using these cost estimates in a back-of-the-envelope calculation of the benefits of the program, we find that HeadStart saved roughly £6 million from avoided exclusions in 2017/18 (the only year where the impact has a p-value below 0.1). While these savings represent about 10% of the overall programme funding, the fact that the reduction in exclusion rates may be down to changes in school policies rather than to changes in student behaviour suggests that a more cost-effective way to reduce exclusions would be for policy makers to change recommendations on exclusions (as is currently done in Scotland) and/or intervene directly with school management stuff to change approaches to discipline.

The second contribution of the paper was to show that synthetic control method is an appropriate methodology to investigate the effectiveness of this intervention. Our results highlighted SCM can be the attractive methodology, especially when there is no obvious control group to make a comparison analysis. As a data-driven procedure, it reduces discretion in the choice of the comparison control groups and allows us to investigate complex area level interventions between the LAs where HeadStart interventions were available and those where the interventions were not available. It increases the objectivity when choosing comparison control units by utilising a data-driven procedure that forces researchers to demonstrate similarities between the treatment and control units. It also does not rely on parallel pre-implementation trends but relies on there being a close pre-treatment match with the donor pool. Lastly, assuming that SCM achieved a good fit over a sufficient period of time in the pre-implementation period, it accounts for both observed and unobserved time-varying confounding that might have an impact on the outcome of interest. We show that we achieve very small RMPSE, indicating an excellent fit between the treated and synthetic control groups.

It is important to note the limitations of the methodology. The main limitation is related to the calculation of statistical significance as it relies on placebo tests rather than the more common approach of standard errors. Secondly, the credibility of the results depends on achieving a good pre-implementation fit between the treated unit and the control unit and there is no consensus on what constitutes a ‘good fit’ or how to judge similarity. For this study, it was easy to achieve a good synthetic control but studies investigating a treated unit that is an outlier may find it hard to find control units that are similar to the treated unit. Lastly, there is not a general agreement on which covariates and pre-treatment periods to be used to achieve a good pre-implementation fit (Bouttell, Craig, Lewsey, Robinson & Popham, 2018).

In conclusion, our findings suggest that large-scale investment in mental health programmes similar to HeadStart has potential for modest reduction in school level outcomes such as exclusions, but it may be difficult to sustain immediate impacts without further effort at keeping momentum or reducing staff turnover. More detailed studies on the mechanisms of effect would also be valuable, though are difficult to conduct in administrative data and without routine collection of mental health data across areas. Such studies would help us to understand how complex programmes can most effectively be implemented to mitigate the negative consequences of exclusions. The findings also show that SCM is a valuable addition to the range of approaches to evaluate an impact of a complex programme when a randomised trial is impractical. Wider use of SCM will help to develop a better understanding of its strengths and limitations.

## CRediT authorship contribution statement

**Sarah Cattan:** Conceptualization, Methodology, Data curation, Formal analysis, Investigation, Software, Writing – original draft, Writing – review & editing, Funding acquisition. **Suzet Tanya Lereya:** Investigation, Software, Writing – original draft, Writing – review & editing. **Yeosun Yoon:** Data curation, Writing – review & editing. **Ruth Gilbert:** Conceptualization, Supervision, Writing – review & editing, Funding acquisition. **Jessica Deighton:** Conceptualization, Supervision, Writing – review & editing, Funding acquisition.

## Figures and Tables

**Appendix Table A1:** Means of variables used in the synthetic control method to creates weights, in the HeadStart LAs and in the synthetic control group.

	% sessions missed for authorised reasons		% sessions missed for unauthorised reasons		% sessions missed b/c fixed term exclusion		% pupils ever excluded		% year 11 pupils with at least 5 GCSEs A*-C	
	HeadStart	Synthetic control	HeadStart	Synthetic control	HeadStart	Synthetic control	HeadStart	Synthetic control	HeadStart	Synthetic control
Female	0.495	0.495	0.495	0.496	0.495	0.493	0.495	0.497	0.494	0.494
FSM	0.170	0.180	0.170	0.178	0.170	0.178	0.170	0.192	0.162	0.185
White	0.808	0.788	0.808	0.753	0.808	0.752	0.808	0.711	0.793	0.826
Black	0.046	0.056	0.046	0.073	0.046	0.097	0.046	0.110	0.050	0.044
Asian	0.072	0.059	0.072	0.065	0.072	0.058	0.072	0.072	0.078	0.059
Chinese	0.003	0.004	0.003	0.005	0.003	0.005	0.003	0.005	0.003	0.003
Other ethnic group	0.071	0.091	0.071	0.105	0.071	0.089	0.071	0.104	0.077	0.073
SEN	0.216	0.214	0.216	0.216	0.216	0.217	0.216	0.220	0.159	0.186
IDACI rank	41.879	42.169	41.879	43.148	41.879	44.102	41.879	40.346	41.762	42.984
Ever CIN	0.042	0.033	0.042	0.034	0.042	0.034	0.042	0.035	0.060	0.054
Ever CLA	0.003	0.003	0.003	0.003	0.003	0.004	0.003	0.004	0.004	0.004
Outcome in 2008/09	0.058	0.058	0.017	0.017	0.002	0.002	0.064	0.064		
Outcome in 2009/10	0.055	0.055	0.017	0.017	0.002	0.002	0.059	0.059		
Outcome in 2010/11	0.051	0.051	0.017	0.017	0.002	0.002	0.056	0.056		
Outcome in 2011/12	0.046	0.046	0.015	0.015	0.001	0.001	0.050	0.050		
Outcome in 2012/13	0.046	0.046	0.014	0.014	0.002	0.002	0.044	0.044		
Outcome in 2013/14	0.041	0.041	0.014	0.014	0.001	0.001	0.046	0.046	0.560	0.563
Outcome in 2014/15	0.042	0.042	0.014	0.014	0.001	0.001	0.049	0.049	0.591	0.594
Outcome in 2015/16	0.039	0.039	0.014	0.014	0.001	0.001	0.050	0.050	0.611	0.614
***Sample size***										
*Individual observations*	*20,682,388*		*20,682,388*		*20,682,388*		*20,682,388*		*20,682,388*	
*Local Authorities*		*149*		*149*		*149*		*149*		*149*

**Appendix Table A2:** HeadStart impacts and pvalues on additional outcomes.

	Impact of Headstart			RMSPE
	2016/17	2017/18	2018/19	
Fraction of pupils excluded for fixed period	−0.0053696	−0.007193	−0.0045551	6.27E-14
	*(0.10)*	*(0.13)*	*(0.25)*	
Fraction of pupils excluded permanently	−0.0003817	−0.0007531	−0.0005684	6.04E-16
	*(0.15)*	*(0.09)*	*(0.26)*	
Fraction of pupils with at least 5 GCSEs A*-C, incl English and Math	−0.0262614	−0.0138997	0.0081832	9.70E-14
	*(0.01)*	*(0.15)*	*(0.37)*	

Note: This first three columns of the table report the estimate of the impact of HeadStart funding and their pvalue (in parenthesis) in each of the first three years of implementation. The fourth column reports root mean squared prediction error (RMSPE).

**Appendix Table A3:** Average impact of HeadStart in the years 2017 to 2019 with associated p-value across students in year 7 to 11, gender.

	2016/17				2017/18				2018/19				RMSPE (computed based on years 2009–2016)	
Outcome Variable	Male		Female		Male		Female		Male		Female		Male	Female
	HeadStart Impact	P value	HeadStart Impact	P value	HeadStart Impact	P value	HeadStart Impact	P value	HeadStart Impact	P value	HeadStart Impact	P value		
% sessions missed for authorised reasons	0.001	0.279	0.000	0.347	−0.001	0.175	−0.001	0.255	−0.001	0.287	0.000	0.398	3.56E-14	7.57E-13
% sessions missed for unauthorised reasons	0.000	0.438	0.000	0.367	0.000	0.458	0.001	0.223	0.000	0.490	0.001	0.279	2.63E-14	3.74E-14
% sessions missed b/c fixed term exclusion	0.000	0.223	0.000	0.267	0.000	0.303	0.000	0.550	0.000	0.291	0.000	0.143	5.27E-16	2.27E-14
% pupils with fixed term exclusion	−0.008	0.131	−0.005	0.028	−0.010	0.159	−0.005	0.159	−0.008	0.243	−0.001	0.426	6.19E-14	2.77E-15
% pupils with permanent exclusion	0.000	0.410	0.000	0.315	−0.001	0.386	0.000	0.450	−0.001	0.402	0.000	0.625	5.27E-17	1.20E-15
% pupils ever excluded	−0.008	0.131	−0.005	0.028	−0.010	0.143	−0.005	0.147	−0.008	0.247	−0.001	0.402	4.71E-14	7.09E-15
% year 11 pupils with at least 5 GCSEs A*-C	0.014	0.127	−0.006	0.438	0.003	0.287	−0.002	0.570	0.031	0.020	0.024	0.028	8.32E-13	3.69E-13
% year 11 pupils with at least 5 GCSEs A*-C, incl. English and math	0.001	0.474	−0.005	0.673	−0.019	0.080	−0.004	0.629	0.031	0.072	0.018	0.024	9.62E-13	6.29E-14
Sample size	1366,779		1330,253		1397,140		1361,305		1434,102		1398,063		11,133,281	10,810,080

**Appendix Table A4:** Average impact of HeadStart in the years 2017 to 2019 with associated p-value across students in year 7 to 11, Special Education Need .

	2016/17				2017/18				2018/19				RMSPE (computed based on years 2009–2016)	
Outcome Variable	No SEN		SEN		No SEN		SEN		No SEN		SEN		No SEN	SEN
	HeadStart Impact	P value	HeadStart Impact	P value	HeadStart Impact	P value	HeadStart Impact	P value	HeadStart Impact	P value	HeadStart Impact	P value		
% sessions missed for authorised reasons	0.001	0.183	0.001	0.554	−0.002	0.100	0.001	0.470	−0.001	0.167	0.002	0.406	4.13E-14	6.84E-13
% sessions missed for unauthorised reasons	0.000	0.406	0.002	0.231	0.000	0.482	0.002	0.303	0.000	0.486	0.001	0.267	1.54E-15	8.55E-14
% sessions missed b/c fixed term exclusion	0.000	0.235	0.000	0.402	−0.001	0.096	0.000	0.335	0.000	0.227	−0.001	0.271	4.31E-14	2.87E-16
% pupils with fixed term exclusion	−0.006	0.048	0.000	0.482	−0.009	0.124	0.002	0.311	−0.007	0.131	0.003	0.299	1.42E-13	3.09E-14
% pupils with permanent exclusion	0.000	0.538	−0.001	0.187	0.000	0.275	0.000	0.367	0.000	0.538	−0.002	0.195	3.23E-17	2.30E-16
% pupils ever excluded	−0.006	0.052	−0.004	0.331	−0.009	0.135	−0.006	0.327	−0.007	0.127	0.000	0.410	4.29E-15	2.30E-13
% year 11 pupils with at least 5 GCSEs A*-C	−0.015	0.084	0.014	0.151	0.002	0.406	0.000	0.574	0.024	0.080	0.001	0.331	1.56E-13	1.81E-13
% year 11 pupils with at least 5 GCSEs A*-C, incl. English and math	−0.016	0.227	0.007	0.267	0.000	0.371	−0.008	0.223	0.022	0.215	−0.008	0.458	1.14E-13	9.78E-14
Sample size	2337,683		359,349		2395,385		363,060		2455,077		377,088		17,519,884	4423,477

Abbreviations: No SEN, No special education need status; SEN, With special education need status.

**Appendix Table A5:** Average impact of HeadStart in the years 2017 to 2019 with associated p-value across students in year 7 to 11, Free School Meal Eligibility.

	2016/17				2017/18				2018/19				RMSPE (computed based on years 2009–2016)	
Outcome Variable	No FSM		FSM		No FSM		FSM		No FSM		FSM		No FSM	FSM
	HeadStart Impact	P value	HeadStart Impact	P value	HeadStart Impact	P value	HeadStart Impact	P value	HeadStart Impact	P value	HeadStart Impact	P value		
% sessions missed for authorised reasons	0.000	0.633	0.001	0.259	−0.001	0.084	0.000	0.434	−0.001	0.239	0.000	0.454	2.43E-12	5.19E-14
% sessions missed for unauthorised reasons	0.000	0.462	0.003	0.068	0.000	0.430	0.003	0.116	0.000	0.518	0.003	0.163	5.62E-14	6.11E-15
% sessions missed b/c fixed term exclusion	0.000	0.275	0.000	0.414	0.000	0.239	0.000	0.323	0.000	0.343	0.000	0.359	1.90E-15	1.28E-14
% pupils with fixed term exclusion	−0.003	0.251	−0.008	0.199	−0.004	0.255	−0.007	0.235	−0.004	0.231	−0.004	0.319	2.09E-14	6.10E-13
% pupils with permanent exclusion	0.000	0.032	0.000	0.434	−0.001	0.020	0.000	0.386	−0.001	0.151	0.000	0.629	1.76E-17	1.42E-16
% pupils ever excluded	−0.002	0.279	−0.009	0.179	−0.004	0.263	−0.008	0.219	−0.004	0.255	−0.004	0.311	1.46E-14	4.42E-13
% year 11 pupils with at least 5 GCSEs A*-C	−0.014	0.116	0.001	0.259	0.001	0.343	0.000	0.434	0.018	0.068	0.000	0.454	1.72E-12	5.19E-14
% year 11 pupils with at least 5 GCSEs A*-C, incl. English and math	−0.018	0.068			−0.005	0.438			0.012	0.251			2.94E-14	
Sample size	2328,827		368,205		2393,945		364,500		2410,254		421,911		18,589,955	3353,406

Abbreviations: No FSM, No free school meal eligibility; FSM, Free school meal eligibility.

**Appendix Table A6:** Average impact of HeadStart in the years 2017 to 2019 with associated p-value across students in year 7 to 11, Child in Need or Child Looked After Status .

	2016/17				2017/18				2018/19				RMSPE (computed based on years 2009–2016)	
Outcome Variable	No CIN or CLA		CIN or CLA		No CIN or CLA		CIN or CLA		No CIN or CLA		CIN or CLA		No CIN or CLA	CIN or CLA
	HeadStart Impact	P value	HeadStart Impact	P value	HeadStart Impact	P value	HeadStart Impact	P value	HeadStart Impact	P value	HeadStart Impact	P value		
% sessions missed for authorised reasons	0.000	0.586	−0.001	0.299	−0.001	0.096	−0.002	0.299	−0.001	0.155	−0.001	0.375	1.42E-12	2.97E-14
% sessions missed for unauthorised reasons	0.000	0.390	0.001	0.167	0.000	0.343	0.003	0.135	0.000	0.398	0.003	0.175	1.66E-15	4.46E-15
% sessions missed b/c fixed term exclusion	0.000	0.394	0.000	0.303	0.000	0.251	0.000	0.223	0.000	0.398	−0.001	0.151	3.48E-16	1.96E-15
% pupils with fixed term exclusion	−0.006	0.068	−0.014	0.084	−0.007	0.104	−0.017	0.064	−0.005	0.227	−0.014	0.096	1.40E-13	9.04E-15
% pupils with permanent exclusion	0.000	0.295	−0.001	0.371	−0.001	0.127	0.000	0.390	0.000	0.394	−0.001	0.295	1.16E-15	9.98E-15
% pupils ever excluded	−0.006	0.064	−0.011	0.127	−0.008	0.100	−0.015	0.076	−0.006	0.203	−0.012	0.124	1.42E-13	1.13E-14
% year 11 pupils with at least 5 GCSEs A*-C	−0.015	0.100	0.004	0.470	0.000	0.602	−0.006	0.355	0.015	0.104	0.035	0.084	2.53E-12	5.31E-13
% year 11 pupils with at least 5 GCSEs A*-C, incl. English and math	−0.016	0.080	−0.020	0.124	−0.003	0.490	−0.017	0.163	0.016	0.187	0.030	0.139	3.97E-14	1.13E-14
Sample size	2499,437		196,261		2508,993		246,757		2525,927		301,910		21,178,993	760,984

Abbreviations: No CIN or CLA, Never had child in need or child looked after status; CIN or CLA, Ever had child in need or child looked after status;.

**Appendix Figure B1:**
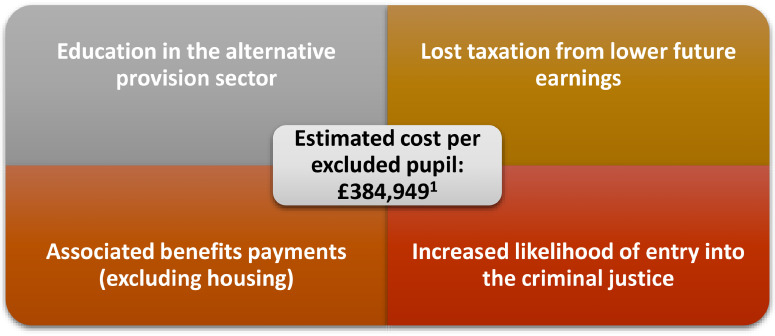
Estimated costs of permanent exclusion^1^.

**Figure:**
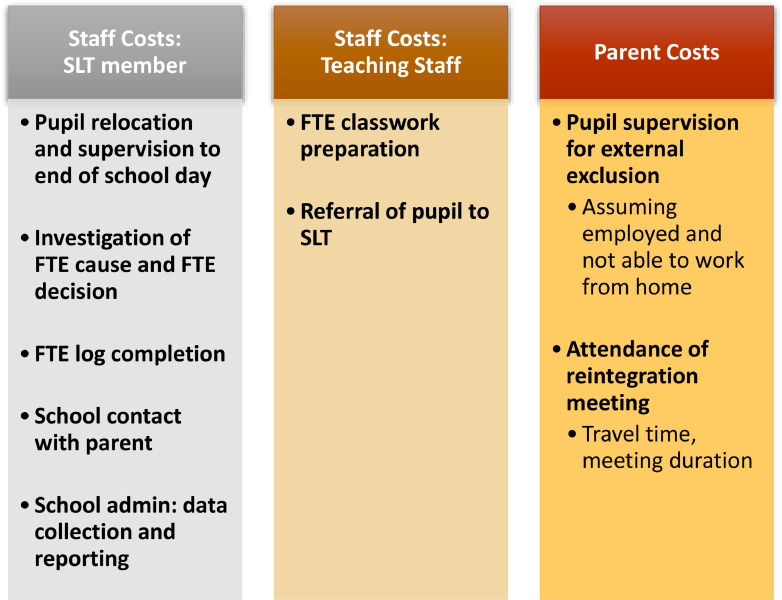


**Appendix Table B1:** Direct Costs.

Cost element	Time required, hours	Unit cost	Cost per internal seclusion	Cost per External FTE	Total FTE costs, England	% of total
Teaching staff						
FTE classwork preparation	0.25	£20.33	£5.08	£2227,183	**£2227,183**	1.7%
Referral of pupil to LT	0.1	£20.33	£2.03	£890,873	**£890,873**	0.7%
LT member						
Pupil relocation and supervision to end of school day	1	£33.36	£33.36	£33.36	**£14,618,650**	11.3%
Investigation of FTE cause and FTE decision	1	£33.36	£33.36	£33.36	**£14,618,650**	11.3%
FTE log completion	0.1	£33.36	£3.34	£3.34	**£1461,865**	1.1%
School contact with parent	0.25	£33.36	£8.34	£8.34	**£3654,662**	2.8%
School admin: data collection and reporting	0.25	£33.36	£8.34	£8.34	**£3654,662**	2.8%
Parent						
Pupil supervision for external exclusion (assuming employed and not able to work from home)	7.5	£21.28	£0.00	£159.60	**£69,948,890**	53.9%
Attendance of reintegration meeting (travel time, meeting duration)	2	£21.28	£0.00	£42.56	**£18,653,037**	14.4%
Total cost				£296	**£129,728,473**	

Abbreviations: FTE, Fixed-term exclusion; LT, Leadership team.

## Data Availability

The authors do not have permission to share data.

## Appendix B Cost estimates for school exclusions

### Permanent Exclusions

The primary quoted cost of permanent school exclusions comes from a 2017 report from the Institute for Public Policy Research,^14^ which estimates a total societal cost of £370,000 for each permanently excluded pupil (assumed to be at 2017 prices, although this is not quoted in the report). Adjustment for the effects of inflation to 2019 prices (the most current available, HM Treasury, 2020), raises this estimate to £384,949.

Using the official figure of 7849 children permanently excluded from school last year, this amounts to £3.02 billion for the 2019 cohort.

Fig. B1 summarises the four key outcomes which are incorporated into the overall cost estimate. Despite detailing the national statistics underpinning the respective cost elements, the report does not specify how the overall cost estimate was calculated, nor what proportion of the overall costs were attributed to each element. The above estimate is almost certainly an underestimate, due to exclusion of several key potential costs. These are:

• Functional exclusion. Pupils educated outside of their mainstream school through offsite alternative provision (in pupil referral units or local authority paid, non-maintained provision). These pupils are not recorded in the official exclusion figures.^1^
• Illegal exclusions. There are no official estimates on illegal exclusions, however 37,500 pupils were not on school roles as of the year 2015/16 and therefore are lost from the safety and education quality oversight.^1^
• Wider costs of involvement in the criminal justice system (CJS) such as victim impact and costs, CJS costs (e.g., courts, probation, prisons etc.) and social services costs for children affected by parental imprisonment.

### Fixed Term Exclusions (FTE)

There are two types of FTEs **Internal Seclusion** and **External Exclusion.** External exclusions typically are 1 to 2 school days, depending on the severity of the cause. FTEs may be due to an accumulation of smaller incidents or from a single triggering incident. Internal seclusions are used as a mechanism to limit external exclusions; for example, to avoid the pupil from being discharged into an unsafe environment.

There is no cost of fixed term exclusion in the literature, so we estimated it based on the following assumptions that are set out below.

#### Assumptions about school procedure

For both FTE to commence, pupils for potential FTE must be referred to a member of the school leadership team (LT). Repeated FTEs (internal or external) may additionally result in the contacting outside agencies (costs not included in this estimate).

#### Assumptions about internal seclusion

• Pupil removed from the classroom and will remain in on-site supervision for the assigned period (typically until the end of the school day)
• Seclusions often result from accumulation of multiple prior warnings
• LT member will investigate the basis for the referral for a formal decision, while the pupil remains under supervision. The time for investigation required may be anywhere between 30 min to a full school day, depending on the nature of the referral
• Teaching staff will prepare class work to be undertaken by the pupil whilst in supervision
• Administrative time will be required which includes the completion of the FTE log (details of referral, contact with parents, follow-up)
• The LT member will contact the parent/guardian to inform them about the details of the seclusion/exclusion and to discuss any actions
• If staff deems appropriate, the pupil will be readmitted to classroom on the following school day

As there are no details of the number of internal seclusions, a national cost estimate of this form of FTE has not been made.

#### Assumptions about external seclusion

For external seclusions, additional actions are required. These are outlined below:

• The LT member will contact the parent/guardian to arrange a readmission meeting
• Reintegration meeting with parents will include parents/caregivers, LT member, staff member or learning mentor (if required). It will last for 10–30 min and there will be a discussion about the requirements for readmission and any additional support needed
• School will collect data on performance and exclusions, and they will be required to periodically report to school governors and Local Authority

**The figure below summarises the cost that we account in our calculation of the cost of a fixed term exclusion:**

**In associating a cost with each of these contributing factors, we further make the following assumptions:**

• Pupils will be excluded for one full school day after referral.
• Teaching staff costs include those for staff actions from time of referral of pupil to Leadership Team (LT).
• Staff costs calculated from minimum teaching staff salary costs for England, excluding London Area.
• Full working day assumed to be 7.5 h.
• Parent/carer assumed to not be required to collect child from school at the time of the referral for FTE.
• Parent is assumed not to able to work from home during pupil exclusion.
• Average national hourly salary for parental time is assumed, accounting for parental age (35 years) and employment rate

Under these assumptions, we estimate that the total cost per FTE is £296. A breakdown of costs for both internal seclusions and external exclusions, are available in Appendix Table B1.

## Footnotes

1 Funding continued to be provided beyond 2018-19, but we cannot estimate the impact of the funding in the subsequent years because data collection was stopped during the pandemic.

2 We analysed 6 local authorities as the intervention sites. However, 12 local areas across England (Middlesbrough, Kent, Lewisham, Newham, Birmingham, Wolverhampton, Cornwall, Hull, Knowsley, Southampton, Blackpool, and Cumbria) were funded to develop local strategies to ‘test and learn’ a whole system approach looking at how a young person's interaction with school, family, community and culture can influence their mental wellbeing in 2013/14. The 12 areas were selected on the basis of high levels of need for preventative mental health services, as well the immense variation between the areas. From 12 local areas, 6 were selected for funding for 5 additional years starting in 2016.

3 The NPD doesn't include data on pupils attending privately funded schools. This represents 7% of all pupils in secondary school in England, but an even lower percentage in the 6 Headstart partnerships which are more deprived than the average LA in England.

4 GCSEs existed well before 2013/14, but data available for this project only included variables that would allow for a consistent definition of the outcome variable from 2013/14 onwards.

5 There was a reorganisation of Local Authorities in 2009, which increased the number of Local Authorities from 150 to 152 by splitting two Local Authorities into two. Because we use data from prior the reorganisation and we need to have a balanced sample across the period of analysis, we use the pre-2009 classification of Local Authorities. We exclude the City of London from the analysis because it is very small and there are many years without data. This yields a sample of 149 Local Authorities, which include 6 Local Authorities with HeadStart funding and 143 in the donor pool.

6 To estimate treatment effect of HeadStart funding in 2016/17, we could have used Wolverhampton in the donor pool. We decided against this in order to keep the LAs in the donor pool the same across the estimation of treatment effects in all three years. Wolverhampton is therefore excluded from the sample when we estimate treatment effects in 2016/17.

7 In additional analyses, we also considered the number of GCSE entries as an outcome of interest. We did not find any impact on this outcome and do not discuss them further here. Results are available upon request.

8 A Child In Need (CIN) refers to a child referred to children's social care, most frequently because of concerns about abuse or neglect, acute family stress or familial dysfunction. A Child Looking After (CLA) is a child whose care has been transferred to the local authority, usually from their birth parents following a child protection investigation. These children are typically accommodated in foster or group homes.).

9 The IDACI measures in a local the proportion of children under the age of 16 who live in low-income households. The local areas for which the index is calculated are Lower Layer Super Output Areas, which represents small areas each with a population of around 1,600 people.

10 Appendix Table A2 reports additional results for fixed term and permanent exclusions separately, as well as for GCSEs including math and English.

11 Stata does not allow us to show all 500 lines corresponding to treatment effects in the 500 placebo tests.

12 For example, analysis by theDfE. (2018) showed that only 1.5% of excluded young people achieved five good GCSEs, including English and Maths. the Youth Cohort Study showed that 27% of excluded pupils were not in employment, education, or training for one to two years when they were 19 years old compared to 10% of non-excluded pupils at the same age (DfE., 2011)

13 Se Appendix B for details about how the cost of each type of exclusions are computed.
